# Association between retinol binding protein-4 and psoriasis vulgaris: a systematic review and meta-analysis

**DOI:** 10.3389/fmed.2023.1208969

**Published:** 2023-08-30

**Authors:** Guangcheng Gao, Yuli Cui, Haiyan Cheng

**Affiliations:** Department of Dermatology, Beijing Friendship Hospital, Capital Medical University, Beijing, China

**Keywords:** psoriasis, dermatology, retinol binding protein, comorbidity, meta-analysis

## Abstract

**Background:**

Psoriasis vulgaris is a chronic skin disease which is related to cardiovascular and metabolic diseases. In the pathogenesis of these diseases, adipokines, including retinol binding protein-4 (RBP-4), play crucial roles. Studies have also shown that RBP-4 might be a meaningful factor in psoriasis however, relying on the analysis of a single study have some drawbacks.

**Objective:**

To evaluate the association between RBP-4 and psoriasis vulgaris more comprehensively.

**Methods:**

Six databases were searched to obtain relevant publications. The selection of the included studies was based on a criteria. The standardized mean difference (SMD) was used for analysis. A value of *p* < 0.05 was defined as significance.

**Results:**

Seven studies were included, with 271 cases and 235 controls. In the comparison between patients and controls, the merged data suggested that levels of RBP-4 were significantly higher in patients (SMD = 0.61, 95%CI: 0.14, 1.07, *p* < 0.05). In five studies containing the data of RBP-4 levels before and after treatment, no significance was found, either for RBP-4 levels in the after-treatment group and control group in these five studies (*p* > 0.05). Subgroup analysis was conducted based on the therapy method. Patients with systematic treatment showed a significant decrease of BRP-4 level after the treatment (SMD = −0.64, 95%CI: −1.26, −0.03, *p* < 0.05).

**Conclusion:**

For patients with psoriasis vulgaris, RBP-4 levels are elevated, and systematic treatment can lower these levels. RBP-4 might act as a key indicator for the diagnosis, efficacy assessment, and comorbidity monitoring of the patients. Further studies with well-designed protocols and enlarged populations are still needed.

## Introduction

Psoriasis is a chronic skin disease associated with the immune system. Worldwide, psoriasis affects about 125 million people. Males and females are equally affected, while adults are more affected compared with children (1, 2). There are different variants of psoriasis, including plaque psoriasis, guttate psoriasis, erythrodermic psoriasis, and pustular psoriasis. With over 80% of the cases, plaque psoriasis, or psoriasis vulgaris, is the most common subtype (1). The specific mechanism of psoriasis is not totally clear, given the complexity of multiple factors involved. Currently, widely recognized risk factors include genetic susceptibility, environmental influence, living habits, and psychological factors (3, 4). Several studies have confirmed that psoriasis is related to cardiovascular diseases (CVD) and a series of metabolic diseases, such as metabolic syndrome, non-alcoholic fatty liver, and type 2 diabetes (5, 6). In North America, the prevalence of CVD in psoriasis patients is 14.3%, while in the general population it is 11.3% (7). In patients with severe psoriasis, the risk of early myocardial infarction is higher than 50% (8). And according to Choudhary’s meta-analysis in 2020, the rate of metabolic disease in psoriasis patients was 30.29% and in general controls it is 21.7% (9). Considering all the current treatments targeting the possible pathogenic factors of psoriasis, few focus on mutually beneficial strategies. Therefore, we looked into these involving pathogenic factors for both psoriasis, CVD, and metabolic diseases.

In the pathogenesis of CVD and metabolic diseases, adipokines play a crucial role in regulation and mediation (10, 11). Retinol binding protein-4 (RBP-4) is one of these adipokines, which belongs to the lipocalin protein family. The main function of RBP-4 is to transport retinol from the liver to target cells or tissue and thus RBP-4 can regulate the metabolism of retinol (12). Additionally, RBP-4 is involved in numerous diseases, including cardiovascular disease, type 2 diabetes, metabolic dysfunctions, and obesity (12, 13). Studies have also found a change in RBP-4 levels in psoriasis patients, indicating that RBP-4 may be a meaningful factor in psoriasis. However, relying on the analysis of a single study can have drawbacks. For example, the sample sizes may be limited, which may lead to low statistical power. Additionally, divergent results may be shown among different studies. Therefore, we performed this meta-analysis, aiming to evaluate the association between RBP-4 and psoriasis vulgaris more comprehensively.

## Materials and methods

### Search of the publications

In total, six databases—PubMed, Ovid, EBSCO, Web of Science, Sinomed, and CNKI—were searched up to 31 January 2022. The words searched for were “Retinol Binding Protein-4” and “Psoriasis.” In PubMed, the search strategy was “((((Retinol Binding Protein-4[Title/Abstract]) OR (Retinol Binding Protein 4[Title/Abstract])) OR (RBP-4[Title/Abstract])) OR (RBP4[Title/Abstract])) AND (Psoriasis [Title/Abstract]).”

### Criteria of selection

We formulated inclusion and exclusion criteria for the selection. Publications meeting the following requirements were included:

- published studies on the relationship between retinol binding protein-4 and psoriasis vulgaris;
- case–control or cohort studies;
- patients with psoriasis vulgaris were included in the case group;
- healthy subjects or subjects without related diseases were included in the control group;
- the level of serum retinol binding protein-4 levels was demonstrated, or enough information was provided to calculate the levels for both groups;
- the method and unit of measurement for RBP-4 detection were provided and
- the language was Chinese or English.

Publications with at least one of the following items were excluded:

- publications from conferences, case series or reports, review articles, commentary articles, animal studies, or graduation theses;
- duplicates of previous publication (s);
- cases with patients with other types of psoriasis, including erythrodermic psoriasis, generalized pustular psoriasis, or psoriasis arthritis; and
- the level of retinol binding protein-4 was not provided or could not calculated.

There were two investigators (GG and YC) who conducted the selection based on the criteria independently. When disagreement occurred, another investigator (HC) would check the selection and make the final decision.

### Data extraction

The following information was extracted from each included study: (1) first author’s name and publication year, defined as Study ID; (2) country where the study was conducted; (3) gender of the subjects in both groups; (4) sample size in both groups; (5) age of the subjects in both groups; (6) treatment methods and duration of the study with comparison before and after treatment; (7) method and unit of measurement for RBP-4 detection; (8) levels of RBP-4 in both groups.

The extraction was completed by two investigators (GG and YC). Then, another investigator (HC) checked the information and made modifications.

### Methodological quality assessment

The types of study were limited to case–control and cohort studies, so the Newcastle–Ottawa Scale (NOS) was applied to evaluate the study quality (14). Assessments of selection, comparability, and exposure are included in the scale. The total score of the NOS is 9; for a single study, if the score was equal to or more than 6, the quality was considered to be high. Two investigators (GG and YC) conducted the evaluation separately. When there was disagreement over specific items, another investigator (HC) would evaluate the quality independently and give the final score.

### Statistical analysis

To evaluate relationships, the standardized mean difference (SMD) was used. In different studies, the units of measurement used in the detection of RBP-4 varied, and the standardized mean difference (SMD) was used to evaluate the relationship, in order to eliminate the influence brought by the measurements and units. The form of Mean ± SD was used in the data combination procedure of analysis. If the original data in the publication was not in the form of Mean ± SD, the methods from Luo and Wan were applied for calculation and transformation (15, 16).

The analysis procedure was conducted using RevMan 5.3 software. The fixed-or random-effects models for data combination were selected on the basis of the heterogeneity between the included studies in the comparison. The I^2^ test and Q-statistical test were employed to test the heterogeneity. With a value of *p* > 0.10 and I^2^ < 50%, no significance was shown in the heterogeneity, thus the fixed-effects model was applied. Conversely, the random-effects model was applied if heterogeneity was found to be significant. For the comparisons with significant heterogeneity, subgroup analysis was introduced to find the source of heterogeneity. Sensitivity analysis was performed to test the influence of a single study to the total effect of synthesis. In this procedure, every study included in the comparison was ignored one by one. If five studies or more were included in a comparison, funnel plots were performed to estimate the publication bias. For all comparisons, a value of *p* < 0.05 was defined as statistical significance (17).

## Results

### Selection of the publications

**Figure 1:**
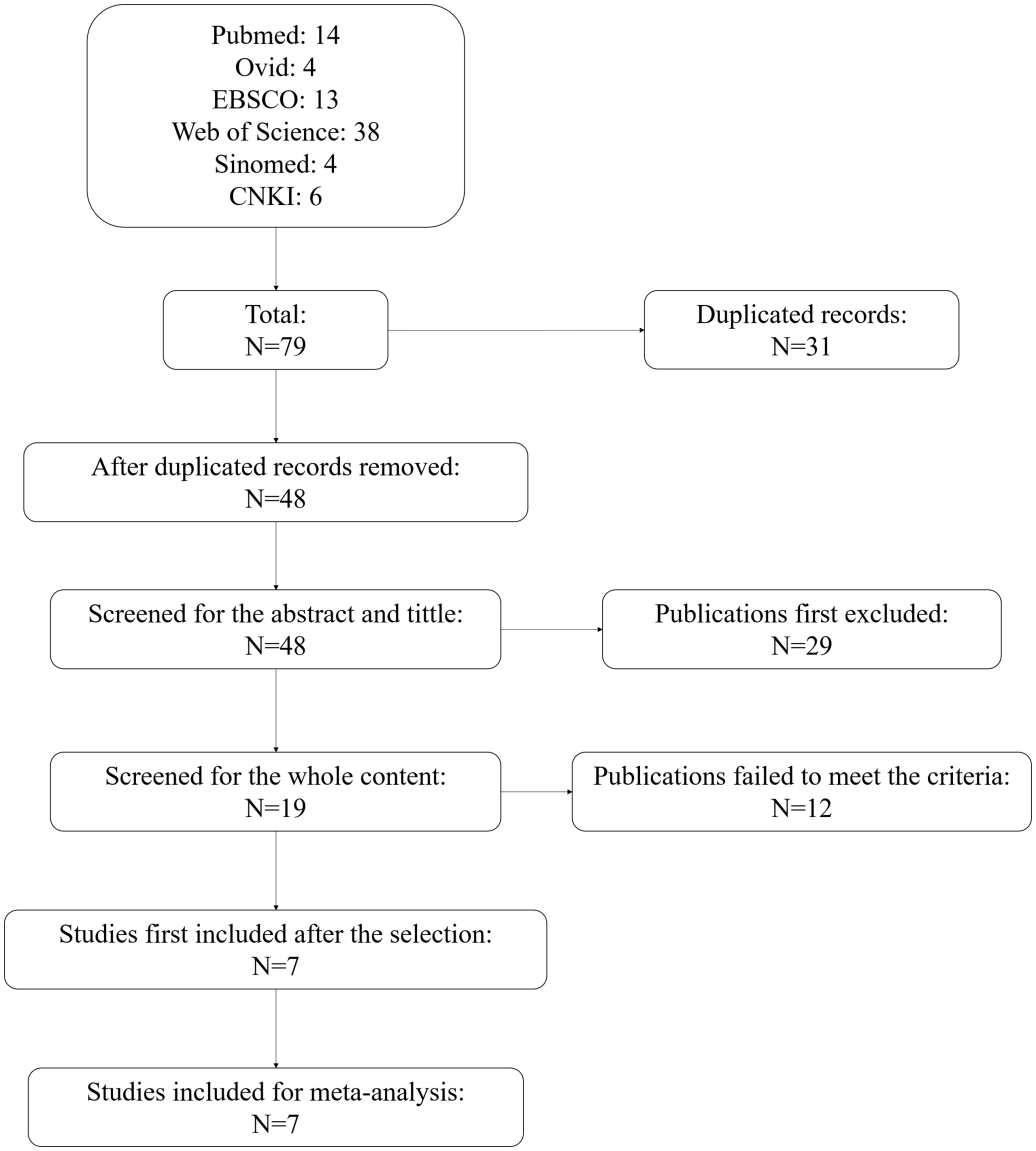
Flowchart of publication selection.

At first, 79 publications from the six databases were obtained. There were 31 duplicated records. After removing these, 48 studies were screened of the title and abstract. In this process, 29 studies were excluded, with 19 checked for whole content. Among these, 12 studies failed to meet the criteria of the selection. Seven studies were first included after the selection. Then, we re-checked the content of the seven studies, and all seven were included for meta-analysis. The flowchart of the selection is shown in Figure 1.

### Quality assessment of included studies

A total of seven studies were included (18–24). The NOS score of the seven studies were 9, 7, 9, 9, 8, 9, and 7, respectively. Since all scores were higher than 6, it was considered they were all of high-quality (Table 1).

**Table 1:** Methodological quality assessment using the Newcastle–Ottawa Scale.

	Karadag et al. (18)	Romani et al. (19)	Baran et al. (22)	Gul et al. (20)	Bakry et al. (21)	Coban et al. (23)	Qiao et al. (24)
**Selection**							
Adequate definition of cases	★	★	★	★	★	★	★
Representativeness of cases	★	★	★	★	★	★	★
Selection of control subjects	★	★	\	★	★	★	\
Definition of control subjects	★	\	★	★	★	★	★
**Comparability**							
Control for important factors	★★	★	★★	★★	★★	★★	★
**Exposure**							
Exposure assessment	★	★	★	★	★	★	★
Same method of ascertainment for all subjects	★	★	★	★	★	★	★
Non-response rate	★	★	★	★	★	★	★
Total score	9	7	8	9	9	9	7

Each ★ stands for one score according to Newcastle-Ottawa Scale.

### Main information of included studies

The studies were published between 2013 and 2019. The case numbers varied from 30 to 50, and a total of 271 cases and 235 controls were included. The locations included Europe (Turkey, Spain, Poland, and Italy), Africa (Egypt), and Asia (China). The methods of detection were all ELISA. Five studies investigated the changes in RBP-4 levels before and after treatment (18, 19, 22–24). The specific treatment methods were provided in four studies (18, 19, 22, 24), while one study included patients with different treatment methods (23). The main characteristics are summarized in Table 2 and Supplementary Table S1.

**Table 2:** Characteristics of included studies.

Study ID	Country	Gender	Number		Age		Treatment		Detection of RBP—4				
			Case	Control	Case	Control	Method	Duration	Method	Unit	Case-before treatment	Case-after treatment	Control
Karadag et al. (18)	Turkey	Both	34	34	Matched		Oral acitretin	3 months	ELISA	ng/mL	295.60 ± 43.10	243.40 ± 36.60	287.60 ± 82.30
Romani et al. (19)	Spain	Both	50	50	46.38 ± 17.29	46.06 ± 17.53	NB-UVB	-	ELISA	ng/mL	15660.86 ± 7,769.53	13991.78 ± 4942.92	10763.52 ± 2,440.74
Baran et al. (22)	Poland	Both	37	16	48.6 ± 2.4	Matched	Topical salicylic acid and anthralin	14 days	ELISA	ng/mL	76787.43 ± 78,655.70	64849.96 ± 67,414.32	70265.99 ± 27,034.45
Gul et al. (20)	Turkey	Both	30	30	33.86 ± 6.6	31.99 ± 6.97	-	-	ELISA	ng/mL	3284.33 ± 1028.87	-	2482.41 ± 446.33
Bakry et al. (21)	Egypt	Both	55	30	40.18 ± 9.08	39.17 ± 9.75	-	-	ELISA	ng/mL	25549.12 ± 9182.79	-	12329.89 ± 2759.18
Coban et al. (23)	Italy	Both	35	50	44.43 ± 11.59	40.48 ± 13.47	Different methods	12 weeks	ELISA	ng/mL	61.01 ± 6.11	64.56 ± 8.83	61.37 ± 6.90
Qiao et al. (24)	China	Both	30	25	Median 36	Median 36	Anti TNF-α biologicals	12 weeks	ELISA	ug/mL	14.91 ± 8.34	11.95 ± 4.47	10.88 ± 6.62

### RBP-4 and psoriasis

**Figure 2:**
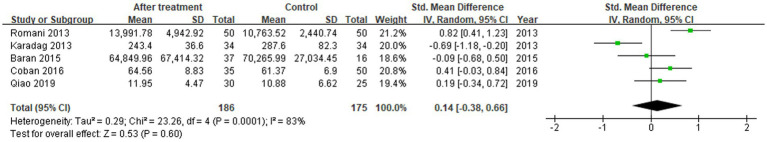
Forest plot of the levels of RBP-4 in psoriasis vulgaris patients and control subjects in seven studies.

All seven studies reported the levels of RBP-4 in patients with psoriasis vulgaris and in control subjects. The heterogeneity between the included studies was significant, so the random-effects model was applied. The merged data suggested that the levels of RBP-4 were significantly higher in patients with psoriasis vulgaris (SMD = 0.61, 95%CI: 0.14, 1.07, *p* < 0.05; Figure 2).

**Figure 3:**
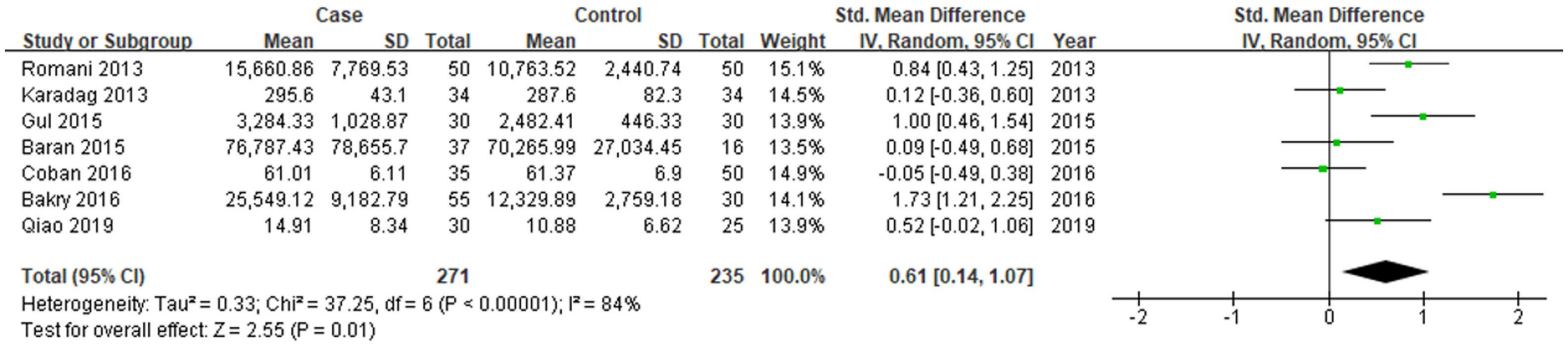
Forest plot of the levels of RBP-4 in psoriasis vulgaris patients after and before treatment in five studies.

Five studies reported the levels before and after treatment (18, 19, 22–24). In the comparisons between the levels after treatment and before treatment, a total of 186 patients were included. The heterogeneity between the studies was significant, so the random-effects model was applied. The merged data suggested that the RBP-4 levels did not change significantly in patients with psoriasis vulgaris after treatment (SMD = -0.33, 95%CI: −0.84, 0.19, *p* > 0.05; Figure 3).

**Figure 4:**
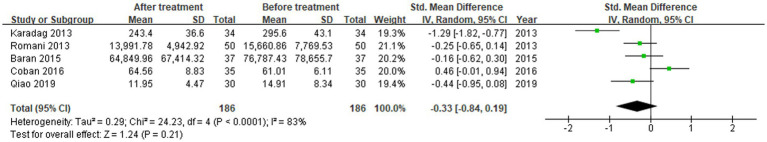
Forest plot of the levels of RBP-4 in psoriasis vulgaris patients after treatment and control subjects in five studies.

In the comparisons between the levels in patients after treatment and those in control subjects in five studies, a total of 186 cases and 175 controls were included. The heterogeneity between the studies was also significant, so the random-effects model was applied. The merged data suggested that the RBP-4 levels in patients with psoriasis vulgaris after treatment were similar to the levels in control subjects (SMD = 0.14, 95%CI: −0.38, 0.66, *p* > 0.05; Figure 4).

### Subgroup analysis

Since the median age of the different studies was very close, and all the included studies’ quality was considered high, we therefore performed subgroup analysis based on gender, duration of treatment, and the location of the patients separately. From this, it turned out that location might be the source of heterogeneity. In the comparison between the levels in cases and in controls, the effects of the Asia and Europe subgroups were different from the total effect. However, the *p*-values of both subgroups were similar to 0.05, with 0.06 in the Asia group and 0.07 in the Europe group. In other comparisons, the effects in different subgroups were all similar to the total effects. Additionally, in the Europe subgroup of all the comparisons, the heterogeneity was lower than that of all the included studies combined, yet it was still significant (Supplementary Figure S1).

In the comparison between the levels before and after treatment, the methods were different, so subgroup analysis was conducted based on topical or systematic therapy. When all the five studies were included, patients undergoing systematic treatment showed a significant decrease (SMD = −0.64, 95%CI: −1.26, −0.03, *p* < 0.05), although no significance was shown in the total effect. Moreover, there was one study that included patients under different treatment methods (20); and it could not be classified to either group. When this study was excluded in the subgroup analysis, a significant decrease in the level of RBP-4 was revealed after treatment (SMD = −0.52, 95%CI: −0.99, −0.04, p < 0.05), meanwhile patients with systematic treatment also showed a significant decrease (SMD = −0.64, 95%CI: −1.26, −0.03, *p* < 0.05; Supplementary Figure S2).

### Analysis of sensitivity and evaluation of publication bias

In the comparison between the levels in cases and in controls, when the 2013 Romani et al. study was ignored, the value of *p* of the total effect was equal to 0.05, with SMD = 0.56, 95%CI: 0.01, 1.12. At the same time, when the other six studies were ignored separately, the value of *p* of the total effect was not altered, which indicated the results were robust and credible in these seven studies.

In the comparison between the levels before and after the treatment, when the 2016 Coban et al. study was ignored, a significant decrease was shown after treatment, with SMD = −0.52, 95%CI: −0.99, −0.04, *p* < 0.05 (Supplementary Figure S3). Since it was different from the value of *p* of the total effect in five studies, this indicated the results in these comparisons were not completely robust. However, the value of *p* was 0.03, which is approximate to 0.05, and when the other four studies were ignored separately, the value of *p* was not altered. Additionally, the sample size in this study was not the largest; therefore, no potential impact was considered on the final results. All three comparisons included at least five studies. The funnel plots were all generally symmetrical, indicating a low publication bias (Supplementary Figure S4).

## Discussion

In this study, we investigated the relationship between retinol binding protein-4 and psoriasis vulgaris through meta-analysis. Our study included seven published studies, comprising 271 psoriasis patients and 235 control subjects. We found that levels of RBP-4 were significantly higher in patients with psoriasis vulgaris, and for patients with systematic treatment, a significant decrease of RBP-4 level was shown. Beyond skin lesions, psoriasis also affects multiple organs or systems such as cardiovascular diseases or metabolic diseases, in which RBP-4 plays vital roles (6, 25). The management of psoriasis has experienced rapid development in recent years, with numerous monoclonal antibodies being applied (26, 27), and better efficacy can be obtained. Furthermore, in the management of psoriasis, the management of comorbidities cannot be ignored. However, current therapy strategies mainly focus on the possible pathogenic factors of psoriasis, and few focus on mutually beneficial factors. Therefore, in this study, we wished to find a potential pathogenic factor both in psoriasis and CVD patients, looking to make a contribution to the understanding of the comorbidities of psoriasis.

The efficacy evaluation of psoriasis is relatively simple to conduct, and is assessed from the changes in plaques and the life quality of patients. However, biomarkers are still needed to assess the underlying inflammation and long-term efficacy, or to predict the efficacy in refractory or severe patients. Additionally, certain indicators can reflect the condition of the patients with comorbidities; for example, a recent study found that gingival crevicular fluid miRNAs from periodontitis patients were associated with cardiovascular disease risk (28). As a result, it is essential to find a biomarker targeting both goals. Generally, researchers have focused on the effects of inflammatory mediators, such as cytokines, chemokines, miRNA, or transglutaminase (28–31). Recently, increasing studies have found the importance of RBP-4 in multiple diseases, including psoriasis. However, due to the limitations of single studies, the results have not yet been good enough, so we used the method of meta-analysis to investigate further.

From the comparison of levels in patients at baseline and in controls in the seven studies, we found that the levels of RBP-4 increased significantly in patients with psoriasis vulgaris compared with control subjects. In previous studies, different results have been shown. For example, Gul et al. (20) and Romani et al. (19) found that RBP-4 concentrations were significantly higher in patients, while Baran et al. (22) and Karadag et al. (18) did not observe significant changes in patients and control subjects. Through meta-analysis, the results from different studies were merged, so a more solid conclusion can be made that levels of RBP-4 were significantly higher in patients with psoriasis vulgaris. RBP-4 may be a participant in the mechanism of psoriasis, for the correlation with PASI (psoriasis area and severity index) scores in some research (19, 32, 33). Additionally, the common comorbidities of psoriasis include insulin resistance, obesity, and metabolic syndrome, in which RBP-4 has been found to be involved; hence, it is rather complicated to expound the specific actions of RBP-4 in psoriasis with current data.

There were five studies included that contained the data of RBP-4 level before and after treatment. Therefore, we compared the RBP-4 levels before and after treatment, and no significance was found. Then, when we compared the levels in patients after treatment to the levels in control subjects in these five studies, no significance was found either. Since the level of RBP-4 was higher than that in controls according to our previous analysis with seven studies, we further conducted subgroup analysis by topical or systematic therapy and found that the levels decreased significantly in patients undergoing systematic treatment compared with before the treatment. Additionally, we excluded the study with mixed methods (23), and significant decreases were shown both in the total effect and in the subgroup with systematic treatment in the new comparison. Therefore, we assumed systematic treatment can lower levels of RBP-4 in patients with psoriasis vulgaris. RBP-4 may be a participant in the genesis of psoriasis, and with the application of systemic treatment, the pathway with RBP-4 involved may be blocked.

Biologic therapy of psoriasis has experienced rapid development in recent years (34). In our study, there was one publication investigating the changes in RBP-4 level after anti-TNF-α biologic therapy (24), but no significant change was shown. The reason for this could be that biologic therapy blocks the targets on the pathways more accurately, and certain markers may not possess interactions with RBP-4. Meanwhile, considering the higher RBP-4 levels in patients, future studies should pay more attention to the functions of RBP-4 in psoriasis. Although there has been some rapid development in the systematic treatment for psoriasis, especially in biologic therapies, topical treatment is still important to psoriasis (35). Through our analysis, no significant change in RBP-4 levels was shown for patients undergoing topical treatment, but there was only one included study with topical treatment applied, so it is hard to draw a conclusion to deny the effect of topical treatment on RBP-4 levels.

In this study, three comparisons were conducted: the levels of RBP-4 in patients at baseline and in controls in seven studies; the levels of RBP-4 in patients after and before treatment in five studies; and the levels of RBP-4 in patients after treatment and in controls in five studies. The heterogeneities were all significant, so subgroup analysis was needed. When we performed subgroup analysis by different region, we found that the levels of RBP-4 were not elevated significantly in patients in Asia and Europe. Region could be a source of the heterogeneity. Among different regions, there might be differences in genetic backgrounds (36, 37), which could have also caused the heterogeneity. Additionally, most of the included studies in Europe investigated the changes after treatment, in which the patients were mainly recruited from clinical trials which were strict with the included patients, some psoriasis patients with severe CVD might be excluded from the clinical trial, thus causing data bias. Different treatment methods may attract and target patients with different manifestations, so differences were shown in the patients among the studies, which caused the heterogeneity. Therefore, further studies with well-designed protocols and enlarged populations are still needed.

Some limitations existed in this study. First, the sensitivity analysis showed that the results were not perfectly robust, and further studies with more cases including the levels before and after topical/systematic treatments are needed. Secondly, the relationship between severity and RBP-4 levels was not analyzed, because the original datasets were not attainable. For the same reason, the relationship between the efficacy and changes was not investigated. Additionally, it would be more beneficial to analyze RBP-4 in psoriasis patients with different comorbidities, especially cardiovascular disease and metabolic diseases. Additionally, in the subgroup analysis based on treatment, further classification of specific treatment methods was not possible to conduct due to the limited studies. In conclusion, larger sample sizes with different treatment strategies with different comorbidities and documenting detailed before/after levels of RBP-4 would help generate more thorough and robust results.

## Conclusion

For patients with psoriasis vulgaris, the levels of retinol binding protein-4 are elevated, and systematic treatment can lower RBP-4 levels. As RBP-4 plays an important role in psoriasis, CVD, and metabolic disease, it might act as a key indicator for the diagnosis and efficacy assessment of psoriasis and especially for the mutual pathogenic factors of the comorbidities in psoriasis patients, which might help contribute to future mutually beneficial treatment strategies. Therefore, further studies with well-designed protocols and enlarged populations are still needed.

## Data availability statement

The original contributions presented in the study are included in the article/Supplementary material, further inquiries can be directed to the corresponding author.

## Author contributions

GG and YC conducted the literature research and data extraction. GG wrote this manuscript. HC designed the study, edited the manuscript, evaluated methodological quality, analyzed the data, and created all the figures and tables. All authors contributed to the article and approved the submitted version.

## Conflict of interest

The authors declare that the research was conducted in the absence of any commercial or financial relationships that could be construed as a potential conflict of interest.

## Publisher’s note

All claims expressed in this article are solely those of the authors and do not necessarily represent those of their affiliated organizations, or those of the publisher, the editors and the reviewers. Any product that may be evaluated in this article, or claim that may be made by its manufacturer, is not guaranteed or endorsed by the publisher.
