# Supplementary figures and images for: Association between retinol binding protein-4 and psoriasis vulgaris: a systematic review and meta-analysis
Source: Front Med (Lausanne). 2023 Aug 30;10:1208969. doi: 10.3389/fmed.2023.1208969 (PMC10498455; doi:10.3389/fmed.2023.1208969)

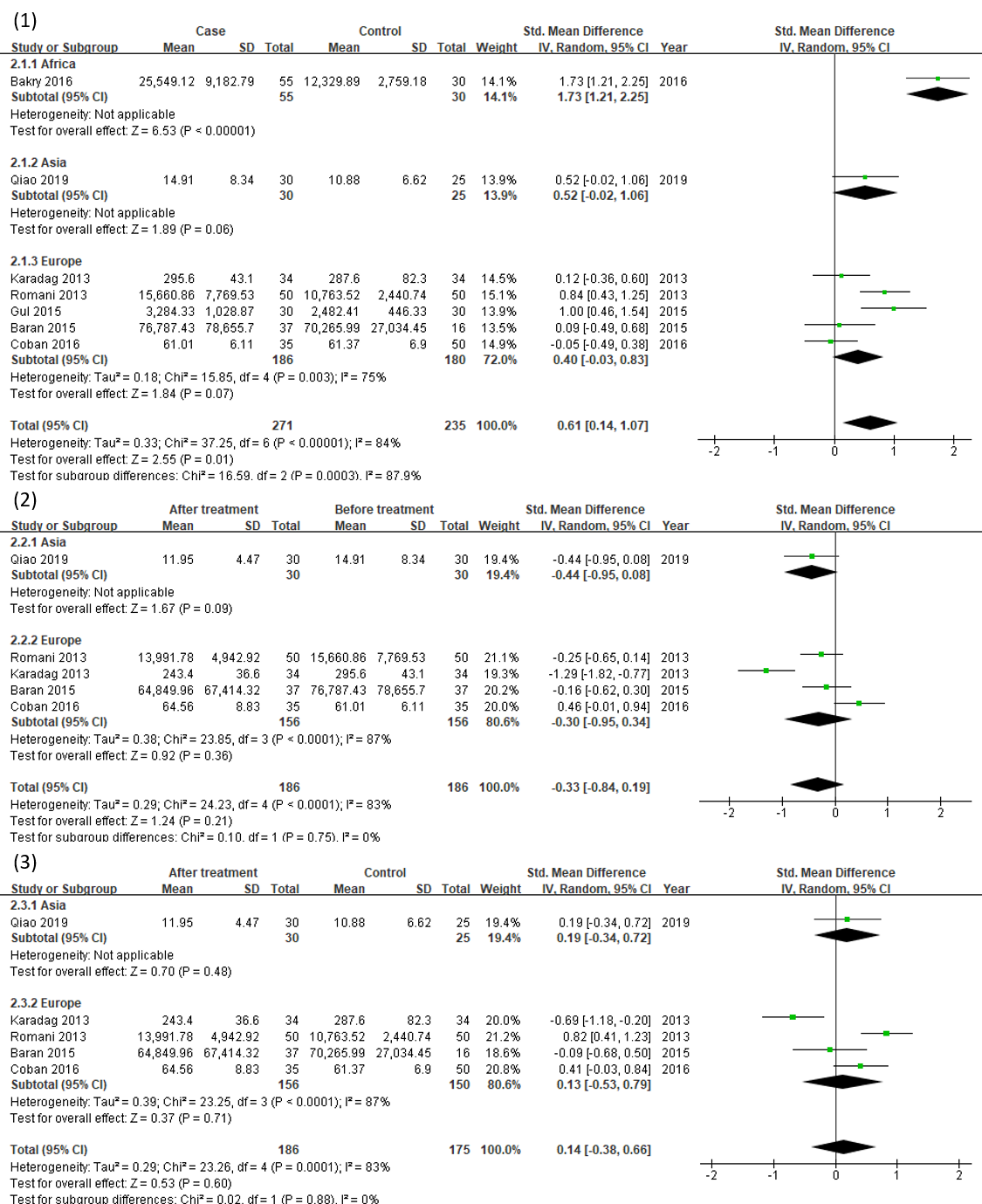

Supplement: Supplementary file 1 [file Image_1.TIF]

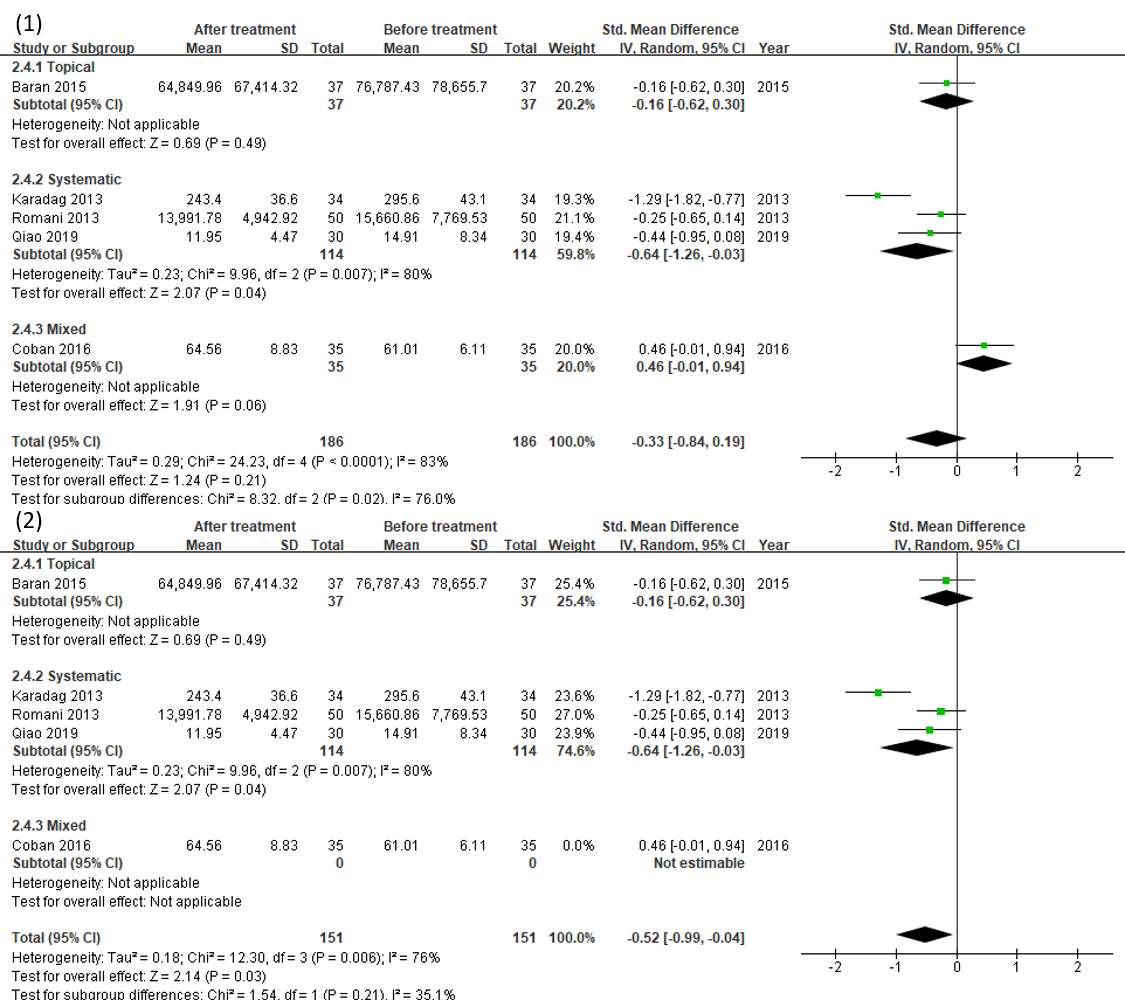

Supplement: Supplementary file 2 [file Image_2.TIF]

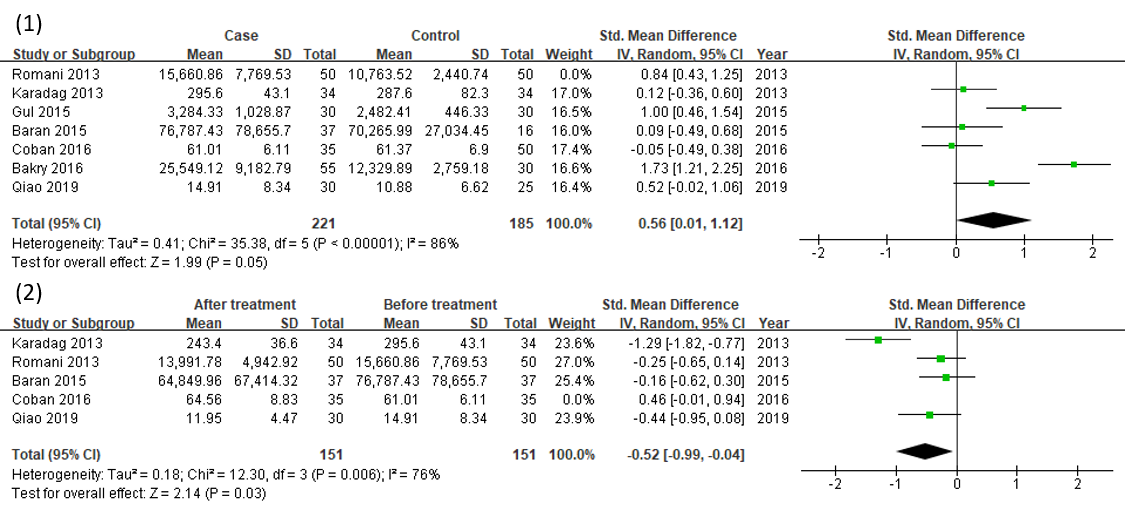

Supplement: Supplementary file 3 [file Image_3.TIF]

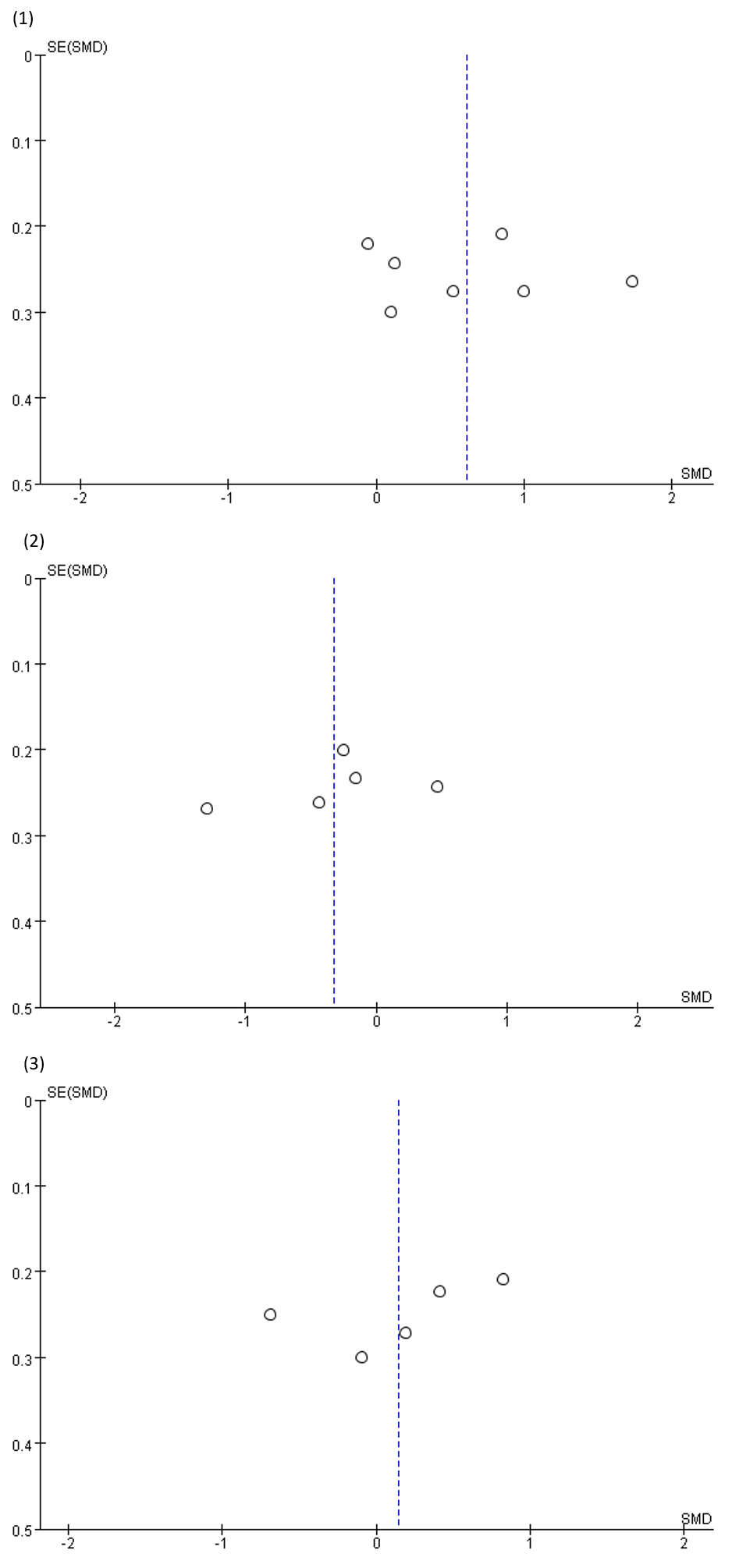

Supplement: Supplementary file 4 [file Image_4.TIF]
