# Supplementary material for: Association between retinol binding protein-4 and psoriasis vulgaris: a systematic review and meta-analysis
Source: Front Med (Lausanne). 2023 Aug 30;10:1208969. doi: 10.3389/fmed.2023.1208969 (PMC10498455; doi:10.3389/fmed.2023.1208969)
Supplement: Supplementary file 5 [file Table_3.DOCX]

sTable 1. Summary of description of control groups

| Study ID | Description of control groups |
| --- | --- |
| Karadag 2013 | Healthy volunteers, matched for age and gender |
| Romani 2013 | Controls matched for gender, age and BMI |
| Baran 2015 | Healthy controls, matched for age and gender |
| Gul 2015 | Healthy volunteers, matched according to BMI and age |
| Bakry 2016 | Age, gender and BMI - matched healthy subjects |
| Coban 2016 | Healthy volunteers of matching age, gender, and BMI |
| Qiao 2019 | Healthy controls |
